# Case of Contraction of the Uterus around the Neck of the Fetus, in Which the Long Forceps, and Ultimately Perforation of the Head, Were Resorted to, for the Safe Delivery of the Mother

**Published:** 1822-04

**Authors:** J. Plumbe

**Affiliations:** Member of the Royal College of Surgeons, &c. &c.


### OBSTETRICAL MEDICINE AND SURGERY.

Art. VI.—*Case of Contraction of the Uterus around the Neck of the Fetus, in which the long Forceps, and ultimately Perforation of the Head, were resorted to, for the safe Delivery of the Mother.* By J. Plumbe, Esq. Member of the Royal College of Surgeons, &c. &c.

SARAH MAUNDY, a stout and apparently robust, healthy woman, about twenty-five years of age, had passed through two difficult and tedious labours within the last four years, and, on September 12th, 1821, was again seized with labour-pains.

On the 14th, the writer was desired to see her; when, on an examination being instituted, the vertex was ascertained to be presenting at the upper orifice of the pelvis. The diameter of the latter, from before backwards, was unusually short, and there appeared to be but little hope that the obstacle thus offered to the passage of the head of the child could be overcome by any efforts which the remaining strength of the mother might be equal to. She was not, however, so completely exhausted as to justify an immediate resort to the operation of cephalotomy. A drachm of laudanum was given, with the hope of procuring her a little rest, and checking the harassing, ineffectual pains by which she was at this moment much distressed.

At 3 P.M., after a short sleep, she was again visited, and, on a second and more minute examination, it was found that the hand, in the absence of pain, could be passed up on the forehead, or occiput, of the child, on either side of the pelvis, without difficulty, though the parietal bones were completely wedged in between the pubis and base of the sacrum. By this step, however, a difficulty of greater and more serious magnitude was ascertained to exist,—a contraction of the body of the uterus round the neck of the child.

The great width of the pelvis admitted of the passage of the hand, on either side, up to the constricted part, and the forefinger could be but just introduced under the band formed by the constriction across the fore-part of the neck of the child. On the opposite side, the constriction was not so complete; but the features of the case were still too serious to justify a sanguine hope of the escape of either mother or child.

In this state of matters, having in view the safety of the child, the Assalini forceps, with Dr. Granville's modification, were applied, and a firm grasp with them obtained, but without any successful result; and, in a consultation held with the latter gentleman, it was agreed that the opening of the head could not, with propriety, be any longer deferred.

This operation having been performed, the anticipated difficulty in drawing the fetus through the constricted part was next to be encountered, and the exertion of a steady tractile effort, continued for some time, was attended by the advance of the shoulders, without that yielding of the uterus to the force employed, which might have been anticipated. The delivery was, however, accomplished without much difficulty, though with the painful feeling of apprehension of mischief to the parietes of the uterus, in dragging the shoulders of the child through its constricted part.

As the uterus did not advance when the extracting force was applied by means of the crotchet to the body of the child, it seems probable that the proximity of the pubis to the base of the sacrum afforded the means of retaining it *in situ;* for there can be but little doubt that it would have so yielded, had no further obstacle to delivery existed than that of the uterine contraction.

The placenta having been removed, the uterus was carefully examined, and found to have sustained no obvious mischief. A large projecting ridge was found on its inner surface, extending round the greater part of its circumference, and lessening the diameter of its cavity considerably at this point, and which, it was evident, must have undergone considerable distension in the passage of the body of the child through it.

The patient did not appear, after delivery, to have suffered so much in point of strength as might have been expected. She took half-an-ounce of castor-oil ; and an injection of gruel was thrown into the vagina, with the view of washing away any loose particles of bone which might possibly have remained in the passage.

15*th.*—A considerable degree of tenderness of the lower part the abdomen; pulse hard, beating 120; the bowels have been twice moved by the castor-oil; skin dry, tongue furred, and a general restlessness. The discharge from the vagina neither more nor less than common. She was bled to the extent of sixteen ounces from the arm, without evident relief. Twelve leeches were applied to the abdomen, and the bowels kept acting, by small doses of sulphate of magnesia exhibited every three or four hours. In the evening of the same day, a general mitigation of symptoms : much less pain; pulse softer, and about 100; a disposition to sleep.

16*th.*—Has slept well during the first, but restless and in pain during the latter part of the night. At 11 A.M. the pain had returned in a considerable degree; the pulse again hard, and 120. Bleeding repeated to the extent of twelve ounces, and the aperient medicine continued; a blister covering the abdomen. In the evening much relieved; complains of great weakness, and is disposed to sleep. The bowels still acting, the aperient was discontinued, and liq. ammon. acet. with vin. antim. substituted for it.

17*th.*—Pulse about 100, soft, and weak; has still a little pain in the abdomen, and a good deal of restlessness. The bowels have been moved twice during the night. Strangury from the effect of the blister. Half-a-drachm of laudanum was ordered to be taken at bed-time, with the diaphoretic, if the restlessness continued.

18*th.*—Has passed a pretty good night. Complains of slight pain in the head ; pulse about 90, and soft ; but still a little pain in the abdomen. Bowels disposed to be constipated ; skin moist. Two drachms of sulphate of magnesia were ordered to be taken every six hours, and the saline medicine continued. The blister to be dressed with cerat. lyttæ.

*19th.*—The symptoms generally improved: pulse natural, skin moist, and bowels acting freely; but still a sensation of soreness referred to the region of the uterus, much increased on pressure. This evening a severe shivering fit occurred, lasting about a quarter of an hour, but not followed by heat or perspiration.

*20th.*—The patient has become tired of the blister, and neglected to have it dressed, as directed. The blistered surface nearly healed, and soreness, increased on pressure, distinctly referable to the uterus.

From this period, the features of the case present nothing remarkable, but the regular occurrence of the shivering in the evening, unattended or followed by any mark of irritation in the system. The appetite good, and the patient thinking herself too well to submit to any further medical treatment. She was lost sight of on the 28th; and, about ten days after, died suddenly in one of her shivering fits, attended with a good deal of pain.

The medical gentleman, under whose charge the patient was placed at the time of her death, had unfortunately no opportunity of examining the body, or, it is probable, that a very important acquisition of information might have been effected; that evidence might have been obtained, which would enable us to draw conclusions as to the extent of power of the uterus (after an irregular contraction of this kind,) of yielding, without mischief, to a distending force; and that a useful principle in the management of such cases might have been brought within our reach.

A fatal case has lately been recorded, where the woman remained undelivered, and where the only obstacle to delivery was a simultaneous contraction of the neck, as well as of the body of the uterus, by which the fetus was locked up in its cavity; and, although so extensive a contraction as occurred in our case, seems to shut out a hope of any means being efficacious, it would be of no small importance to know how far we can venture to use force in forwarding delivery in cases approaching to it in similarity, though not in extent.

It appears that, though the means afforded by the lancet of subduing irregular action of the uterus in cases somewhat similar are frequently, and indeed generally, efficacious; yet that bleeding, carried to the greatest possible extent, possesses not that influence in subduing muscular power, as when it is supported by the combined effect of small nauseating doses of the tartarized antimony. By the judicious exhibition of this medicine, the contractile power of the muscles is reduced in an extraordinary manner, and the whole energies of the system laid prostrate. If, therefore, the contracted uterus will, in any state of the system, bear great distension, it must surely be in this; and, when this latter object must be accomplished, to give the patient any chance of life, there can be no hesitation in pronouncing that this medicine ought always to be had recourse to.

It fell to the lot of the writer, some six months since, to be called to a case where the woman had been four days in labour, and was not then delivered. A gentleman, who taught and practised midwifery at that period in London, had been in attendance at the commencement. It was an arm presentation: the child could not be turned, and the arm was cut off. This step, however, was not followed by others having the same object; and, on the third day, several other attempts appear to have been made to turn, without success. On the morning of the fourth day, it was stated that the rigidity of the uterus still prevented a possibility of carrying up the hand. At the period when our first visit was made, (about the middle of the fifth day,) the woman seemed to be quite exhausted, no pains having been for some time felt; and, on the attempt being now made to turn by ourselves, the operation was effected, and the mutilated remains of the child brought away, without the slightest difficulty.

This woman died soon after her delivery; and if, as there is reason to suppose, the emetic tartar, in combination with bleeding, will produce a temporary effect on the muscular system, approaching in similarity to that state when, in the above case, the uterus lost its contractile energies, it promises to be of incalculable advantage in such formidable cases as that recorded by BATTZELL, and that which forms the subject of the present paper.

